# Conformational Sampling of CPT1A Enables Dynamic Structure-Based Ligand Screening

**DOI:** 10.3390/ijms27198840

**Published:** 2026-10-03

**Authors:** Sijia Zhang, Yingbo Zhang, Jiasheng Zhao, Jiangang Long, Lei Zhang, Zhiwei Yang

**Affiliations:** 1 MOE Key Laboratory for Nonequilibrium Synthesis and Modulation of Condensed Matter, School of Physics, Xi’an Jiaotong University, Xi’an 710049, China; zhangsijia@stu.xjtu.edu.cn (S.Z.); zhaojiasheng@stu.xjtu.edu.cn (J.Z.); zhangleio@xjtu.edu.cn (L.Z.); 2 School of Life Science and Technology, Xi’an Jiaotong University, Xi’an 710049, China; zhangyb1124@163.com (Y.Z.); jglong@xjtu.edu.cn (J.L.)

**Keywords:** CPT1A, conformational dynamics, ligand binding, Markov state model, molecular dynamics simulation, lipid metabolism

## Abstract

Carnitine palmitoyltransferase 1A (CPT1A) catalyzes a rate-limiting step in fatty acid β-oxidation and is a target for studies of metabolic disease and cancer. However, the structural dynamics of CPT1A remain poorly understood due to the lack of full-length structure. In this study, we employed an integrated computational approach combining molecular dynamics (MD) simulations, Markov state models (MSMs), and binding free energy calculations to elucidate the conformational landscape of human CPT1A. We performed an all-atom MD simulation of CPT1A embedded in a realistic outer mitochondrial membrane, and root-mean-square fluctuation (RMSF)-guided local region analysis coupled with MSM transition-path analysis and GROMOS clustering convergently identified a representative conformation. The pathway probabilities reported here describe dominant local transitions within specific mobile segments rather than the global equilibrium population of the full-length protein. Using this representative conformation, we performed a virtual screening of over 1.23 million compounds from the ChemDiv library, followed by Lipinski filtering, yielding five candidates with docking scores below −10 kcal/mol. Subsequent MD simulations and binding free energy calculations confirmed stable binding, with Compound **2** exhibiting the most favorable free energy of −25.99 kcal/mol. Principal component analysis (PCA) and secondary-structure analyses revealed ligand-specific modulation of CPT1A dynamics. A preliminary in vitro CPT1 activity assay revealed candidate-specific responses, and the free-energy and activity data were partially consistent. These results provide a dynamically informed structural basis for CPT1A ligand recognition, identify candidates with distinct energetic and functional profiles, and establish an integrated strategy for ligand discovery in structurally uncharacterized proteins.

## 1. Introduction

Recent studies have focused on dysregulated lipid metabolism in obesity and related cardiometabolic complications [1], highlighting carnitine palmitoyltransferase 1A (CPT1A). CPT1A is located in the outer mitochondrial membrane (OMM), where it catalyzes the transfer of long-chain acyl groups from acyl-CoA to carnitine. This process produces acylcarnitine and enables mitochondrial β-oxidation to proceed [2,3,4,5]. Allosteric inhibition by malonyl-CoA makes CPT1A a metabolic rheostat that couples nutrient availability to energy production. Dysfunction of this regulation has been causally linked to non-alcoholic fatty liver disease, type 2 diabetes, heart failure, and metabolic reprogramming in multiple cancers [2,3]. CPT1A is therefore an attractive target for pharmacological modulation, but existing modulators have suboptimal properties, motivating the development of more efficacious agents [2,3].

Human CPT1A is a membrane protein with a short N-terminal regulatory segment, two transmembrane helices, and a large C-terminal catalytic domain [6,7,8,9]. The N-terminus controls malonyl-CoA sensitivity and responds to the surrounding lipid environment. By contrast, the C-terminal region contains the catalytic machinery and most of the predicted ligand-binding cavities. Despite its biological importance, no high-resolution experimental structure of the full-length protein has yet been determined. Predicted models from AlphaFold and related tools provide a workable starting model, but these static snapshots cannot reproduce membrane-induced rearrangements, surface-loop mobility, or the conformations accessible under physiological conditions. In particular, the N-terminal peptide and the linker between the two transmembrane helices show high flexibility, while the active-site pocket may undergo induced-fit changes upon ligand binding. Hence, a static model alone is thus inadequate for rational drug design and for understanding the allosteric regulation of CPT1A protein.

Molecular dynamics (MD) simulations have emerged as a powerful tool to investigate protein dynamics at atomic resolution, because it explicitly includes protein, lipids, water, and ions over time, making it particularly suitable for membrane-protein systems [10,11]. MD trajectories can show how the lipid bilayer affects helix tilting, loop fluctuations, and the opening and closing of substrate channels. A typical MD run, however, produces thousands of frames, and selecting a single representative receptor that is both structurally stable and functionally relevant for docking remains a challenging task. Conventional global clustering tends to overemphasize the rigid core while underrepresenting local motions near the binding site. To address this limitation, time-lagged independent component analysis (TICA) and Markov state models (MSMs) offer a kinetic perspective by highlighting slowly interconverting states that often govern ligand recognition and allosteric transitions [12,13,14]. These methods make it possible to extract metastable conformations and transition pathways, providing a more rational basis for receptor selection than purely geometric clustering.

In this work, we conducted an all-atom MD simulation of CPT1A embedded in a realistic outer mitochondrial membrane. Using RMSF analysis as a guide, we applied local MSM transition-path analysis and GROMOS clustering to five mobile regions, selecting a late-stage apo conformation as the docking receptor [15,16]. This conformation was then screened against 1.23 million ChemDiv compounds with AutoDock Vina 4.2.6, and the resulting hits were assessed for drug-likeness [17,18,19,20,21]. Five candidates were further examined using replicate protein–ligand MD simulations, principal component analysis (PCA), secondary-structure analysis and MM/PBSA binding free-energy calculations. These findings may help clarify the structure–function relationships of CPT1A and inform the development of new therapeutics for metabolic diseases and cancer. They also offer a framework for dynamic conformational sampling and ligand discovery in proteins.

## 2. Results

### 2.1. Structural Stability of Apo CPT1A

**Figure 1:**
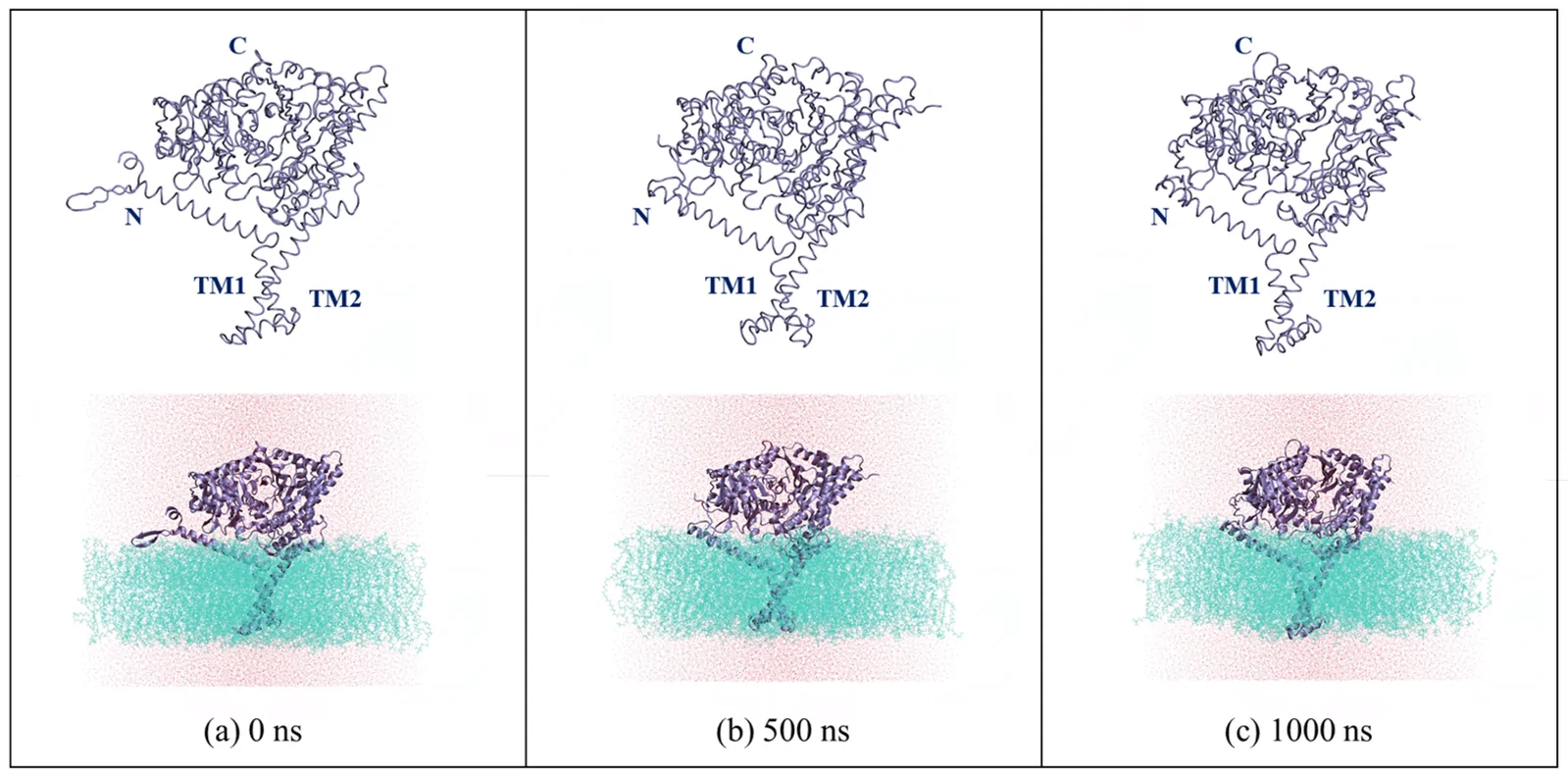
Structural evolution of membrane-embedded CPT1A (apo state) during the 1000 ns MD simulation. CPT1A (purple) and the OMM-mimetic bilayer (teal) are shown at 0, 500, and 1000 ns.

**Figure 2:**
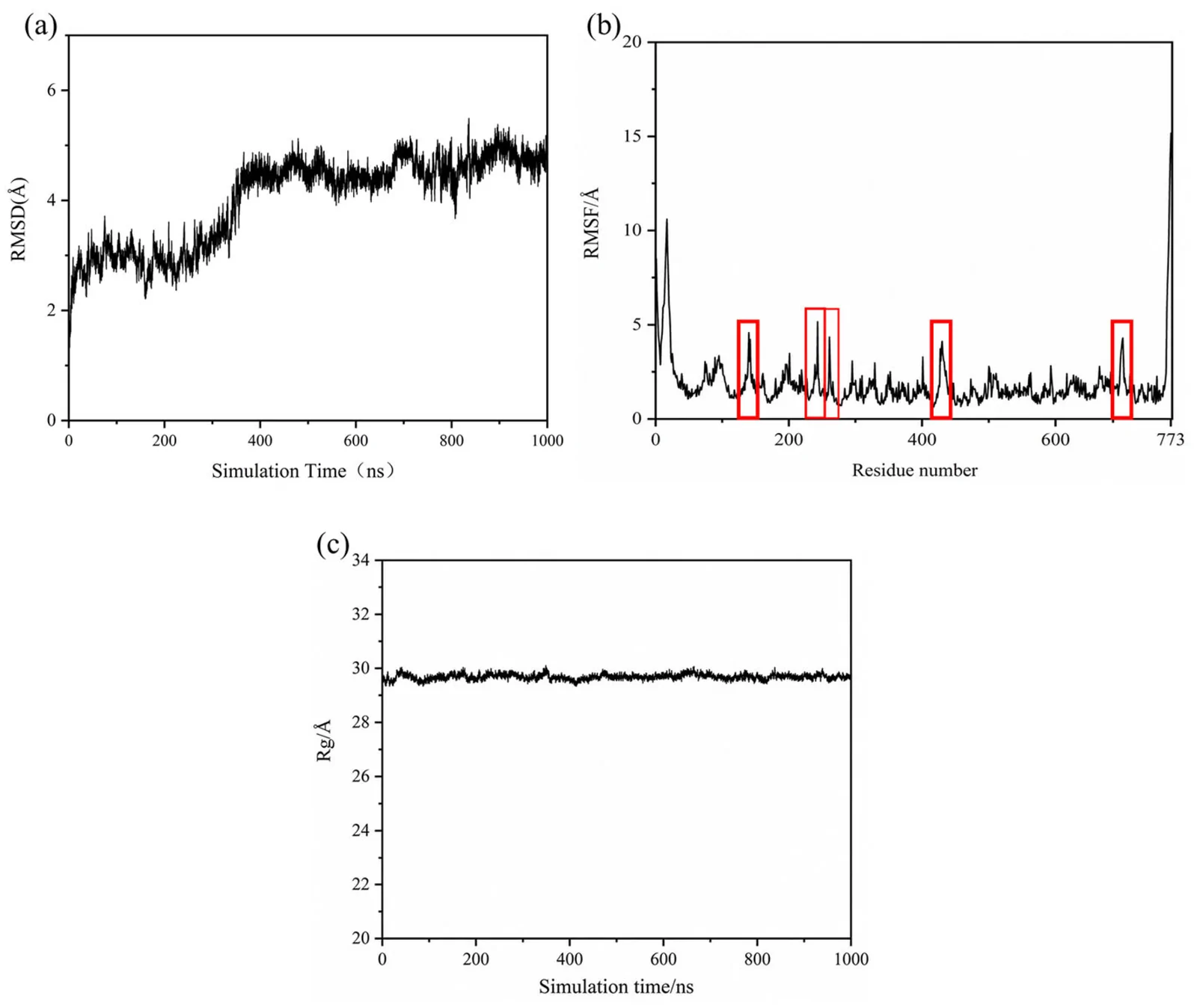
Structural stability and flexibility of membrane-embedded CPT1A during the 1000 ns MD simulations. (**a**) Backbone RMSD. (**b**) Residue-wise RMSF, with five key regions highlighted by red boxes. (**c**) Total radius of gyration (Rg).

To investigate the structural changes in the CPT1A protein in a membrane environment, we performed a 1000 ns simulation of the AlphaFold-derived apo model in an outer mitochondrial membrane mimic. Snapshots taken at 0, 500, and 1000 ns revealed rearrangements in the N-terminal region (Figure 1). Backbone RMSD increased during roughly the first 400 ns and then showed fluctuations of less than ~2 Å over the final 600 ns (Figure 2a). For local MSM analysis, 501 frames were extracted at 2.0 ns intervals from the trajectory.

RMSF values for most residues ranged from 0 to 5 Å (Figure 2b). The largest fluctuations occurred at exposed termini and surface loops, prompting us to select five local regions for MSM analysis. The total Rg remained between 29 and 30 Å across the trajectories, indicating that overall compactness changed little (Figure 2c).

### 2.2. Local Conformational Analysis

Five regions with elevated RMSF were selected to focus the local MSM and structural clustering analyses on mobile segments of the sampled apo trajectory. These regions correspond to residues 132–147 (region 1), 235–247 (region 2), 257–267 (region 3), 420–440 (region 4), and 695–707 (region 5). Each region was then coarse-grained using Perron cluster analysis (PCCA+) into four macrostates, displaying two to four transition pathways. Figure S1 shows the conformations of these four macrostates for regions 1–5. The dominant pathways from macrostate 1 to macrostate 4 were 1 → 3 → 4 in region 1, 1 → 4 in region 2, 1 → 2 → 3 → 4 in region 3, 1 → 4 in region 4, and 1 → 3 → 4 in region 5. Their pathway fluxes were 56.8%, 68.1%, 68.9%, 65.2%, and 75.8%, respectively (Table 1).

**Figure 3:**
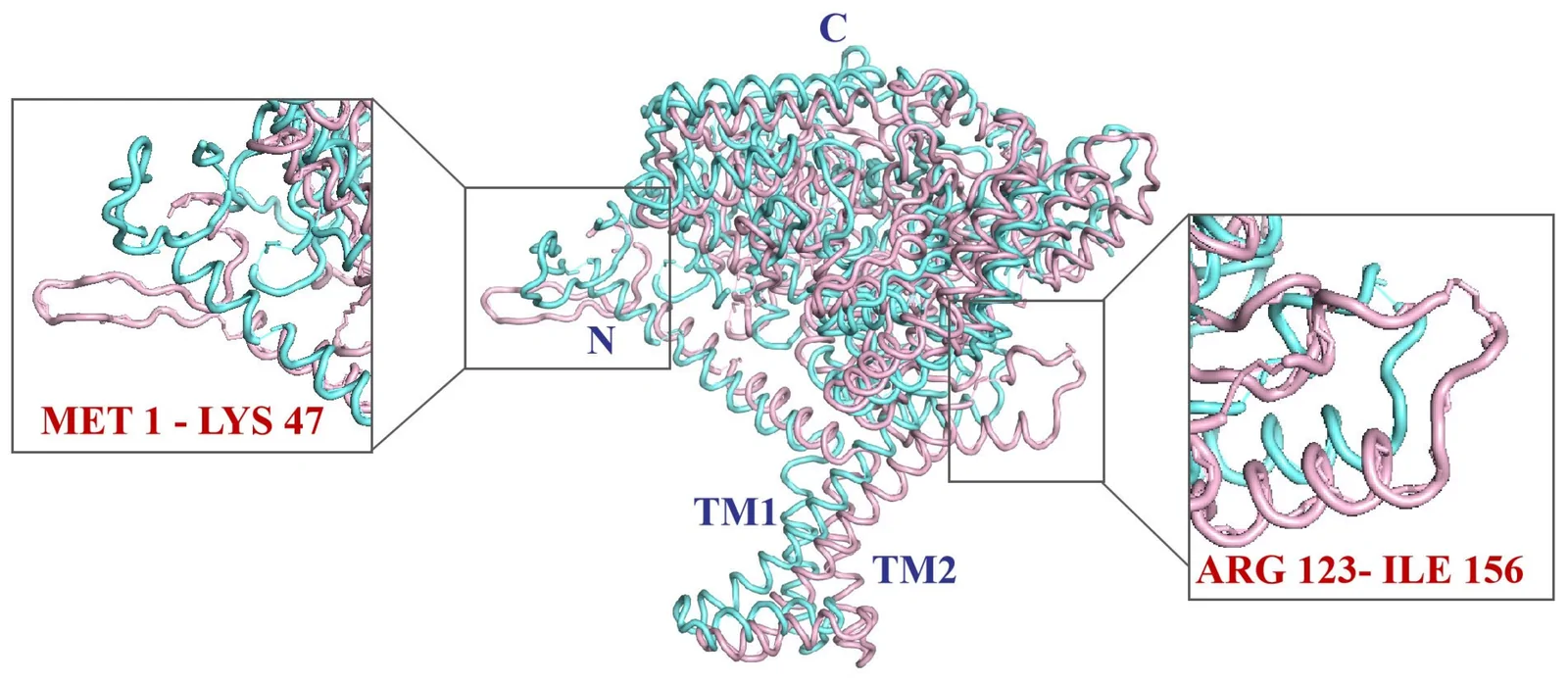
Superposition of the initial CPT1A model (pink) and frame 467 (cyan). The largest rearrangements occur in the N-terminal regulatory segment (Met1-Lys47) and the transmembrane-to-catalytic connector (Arg123-Ile156), while most of the C-terminal catalytic domain remains stable.

GROMOS clustering produced three or four clusters per region (Table 1 and Figure S2). Frame 467 was selected as the initial conformation for the docking from the late-stage portion of the apo trajectories, where the backbone RMSD displayed a plateau (Figure 2a). Superposition with the initial AlphaFold model revealed significant differences in the N-terminal regulatory segment (Met1-Lys47) and the transmembrane-to-catalytic connector (Arg123-Ile156), while most of the C-terminal catalytic domain retained a similar arrangement (Figure 3). Characterization of the selected frame revealed hydrophobic packing, intramolecular contact networks, and predicted cavities in the C-terminal region, providing a structural basis for its use in ligand screening.

### 2.3. Representative Conformation for Docking

To characterize the initial conformation for the docking, we examined its packing, intramolecular contacts, and predicted cavities. ProteinTools detected 26 hydrophobic clusters in frame 467 with interfacial areas ranging from 63.16 to 2109.25 Å^2^. Cluster 16 was the largest (2109.25 Å^2^) and contained 46 contacts, consistent with a densely packed hydrophobic core. We identified thirty-one salt-bridge clusters, and charged residues represented approximately 23% of the sequence, giving a charge-segregation parameter (κ) of 0.19. This indicates that positive and negative charges are relatively well mixed along the chain. Additionally, eighteen hydrogen-bond networks were detected with donor–acceptor distances of 2.49–3.43 Å and donor–hydrogen–acceptor angles of 123.42–178.18°. The contact map also showed long-range contacts between residues far apart in the sequence, supporting the structural integrity of the representative receptor.

**Figure 4:**
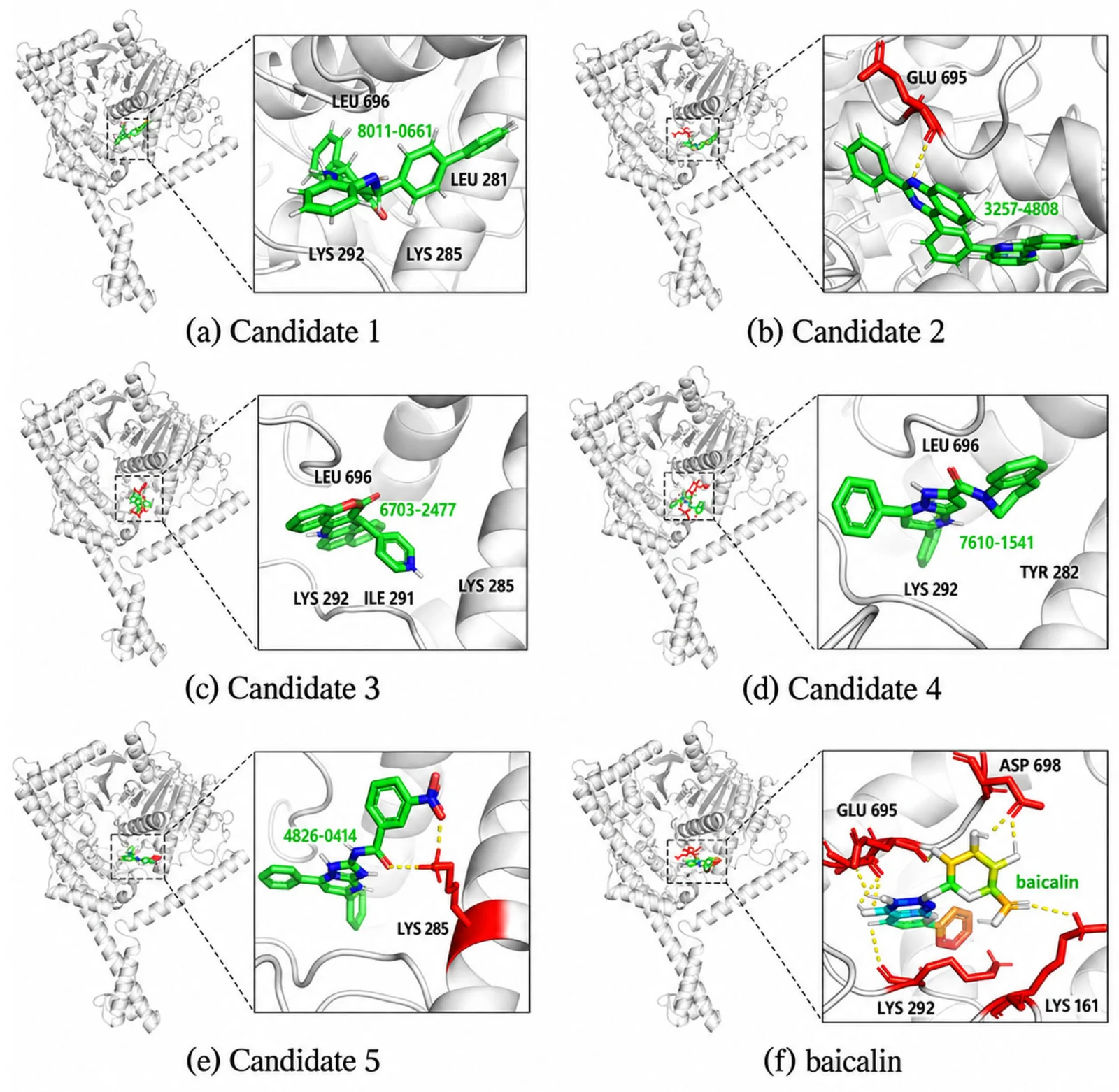
Predicted binding modes of candidates 1–5 (**a**–**e**) and baicalin (**f**) within CPT1A. Each panel shows the overall protein structure and an enlarged view of the binding site. Candidates 1–5 are shown in green, and residues involved in predicted hydrogen bonds are highlighted in red. Yellow dashed lines indicate predicted hydrogen bonds, involving Lys161, Lys292, Glu695, and Asp698 in the baicalin pose.

ProteinsPlus and CavityPlus 2022 predicted 25 and 21 cavities, respectively (Figure S3a,b). Their highest-ranked cavities were concentrated in the C-terminal catalytic region and had surface areas of 2880.07 and 2408.50 Å^2^. Baicalin, a reported CPT1A activator that was docked as a positive reference, formed four hydrogen bonds in its docked pose involving Lys161, Lys292, Glu695, and Asp698 (Figure 4f). These complementary observations defined a C-terminal screening volume that included the Glu695/Asp698 region and was large enough to accommodate ligand rearrangement (Figure S3c).

### 2.4. Docking and Drug-Likeness Filtering

We screened the ChemDiv library against the selected receptor and evaluated the drug-likeness among the top-ranked compounds. Among the approximately 1.23 million screened compounds, 498 had Vina scores below −10 kcal/mol, including 42 compounds below −11 kcal/mol. The 200 highest-ranked compounds from the complete docking-score ranking were subsequently subjected to SwissADME analysis, leading to the selection of five candidates (Table 2). All five candidates had more favorable docking scores than baicalin (−8.26 kcal/mol). Candidate 1 ranked first by this score (−11.2 kcal/mol), followed by candidate 2 (−11.0 kcal/mol). Candidate 2 formed a predicted hydrogen bond with Glu695, a residue also contacted by baicalin, whereas candidate 5 contacted Lys285. No hydrogen bonds were found in the selected poses of candidates 1, 3, and 4 (Figure 4).

Figure S6 compares their two-dimensional structures. The candidates and baicalin share aromatic ring systems and heteroatom-containing functional groups. Baicalin contains a hydroxylated flavone core that is linked to a glucuronic acid moiety. Candidates 1–5 contain nitrogen-containing heterocyclic scaffolds with distinct ring connectivity and substitution patterns; none contains the glucuronide moiety.

### 2.5. Structural Behavior of the Candidate Complexes

**Figure 5:**
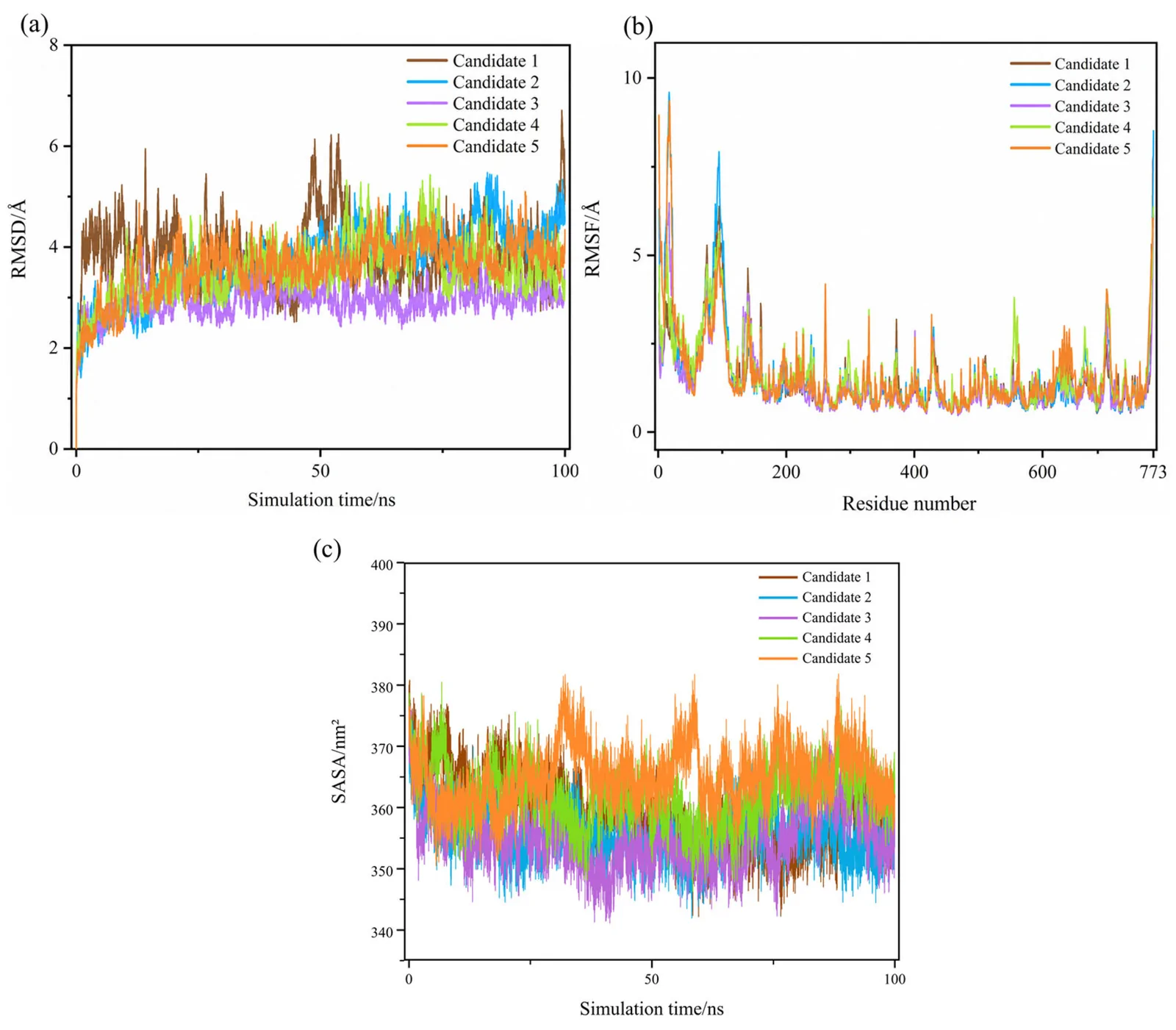
Protein structural behavior in CPT1A complexes with candidates 1–5 during 100 ns MD simulations. (**a**) Backbone RMSDs. (**b**) Residue-level RMSF profiles. (**c**) Solvent-accessible surface area (SASA).

To characterize the candidate complexes during the MD simulations, we monitored protein RMSD, residue-level fluctuations, compactness, and solvent exposure across three independent simulations per complex. Protein RMSD values ranged from approximately 1 to 7 Å (Figure 5a). Candidate 1 showed the largest excursions, including an increase near the middle of the trajectory, whereas candidate 3 remained near 3 Å after its initial adjustment. Candidates 2, 4, and 5 showed broader fluctuations during the sampled interval.

Residue-level RMSF was highest in the N-terminal/transmembrane region (residues 1–122), reaching approximately 10 Å in some trajectories, whereas residues 123–773 generally remained within approximately 3 Å (Figure 5b). Rg values ranged from 28.5 to 30 Å (Figure S4), and SASA values ranged from 340 to 380 nm^2^ (Figure 5c). Thus, the elevated backbone RMSD coexisted with limited changes in overall compactness and solvent exposure, while the largest residue-level fluctuations were concentrated in the N-terminal region.

### 2.6. Conformational Sampling and Secondary Structures

**Figure 6:**
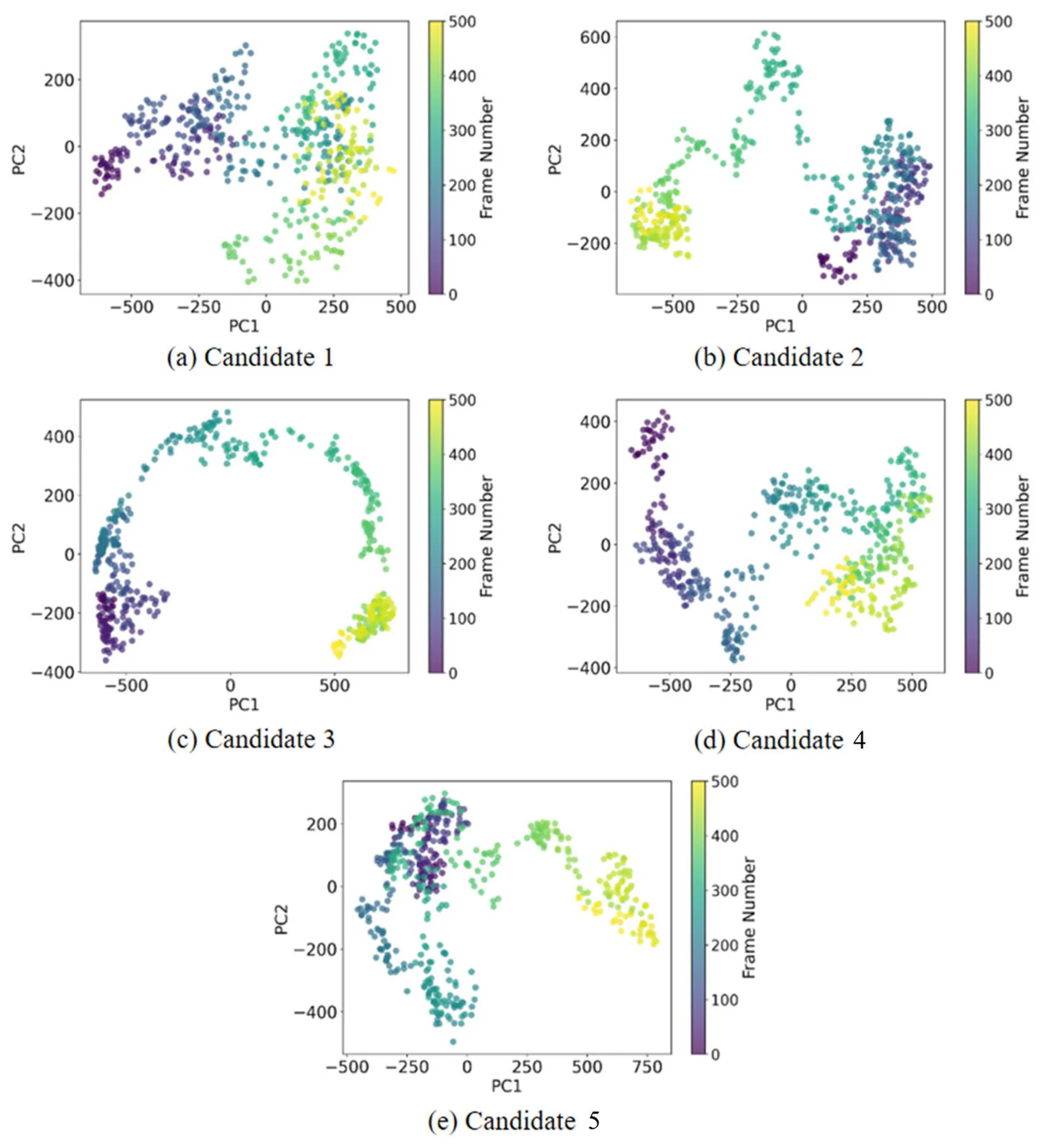
PCA projections of CPT1A C_α_ trajectories for complexes with candidates 1–5 (**a**–**e**). Color gradients indicate simulation progression from early to late frames.

To characterize coordinate variation within each complex, we analyzed independently calculated PCA projections together with secondary-structure evolution. The first two principal components explained 84.63%, 85.13%, 85.86%, 85.99%, and 81.73% of the coordinate variance for candidates 1–5, respectively. In their displayed projections, candidates 1 and 4 occupied two major regions, candidate 2 occupied three partially separated regions, and candidate 3 followed a curved progression from early to late frames. Candidate 5 also exhibited a continuous progression (Figure 6). These projections illustrate the temporal organization of the sampled protein coordinates.

DSSP analysis showed that α-helices remained the predominant secondary-structure element in all complexes, with persistent helical segments spanning residues 1–200 and 600–650 and stable β-structure around residues 300–360 (Figure S5). The sampled complexes retained their main secondary-structure elements, with local transitions visible along the trajectories.

### 2.7. MM/PBSA Estimates for Candidates 

We used MM/PBSA estimates to compare the computed energetic contributions of the candidate complexes (Table 3). All five mean estimates were below −10 kcal/mol, and four were more negative than the baicalin estimate of −13.28 ± 0.42 kcal/mol. Candidate 2 had the most favorable mean value (−25.99 ± 1.85 kcal/mol). Candidates 4 and 1 gave −18.99 ± 1.66 and −18.36 ± 1.40 kcal/mol, respectively, followed by candidates 3 and 5 at −15.47 ± 1.05 and −11.01 ± 3.42 kcal/mol. Van der Waals interactions provided a major favorable contribution for candidates 1 and 2. Candidate 5 displayed the greatest reported variability. Overall, these approximate energy estimates support computational prioritization.

### 2.8. Preliminary In Vitro Evaluation

**Figure 7:**
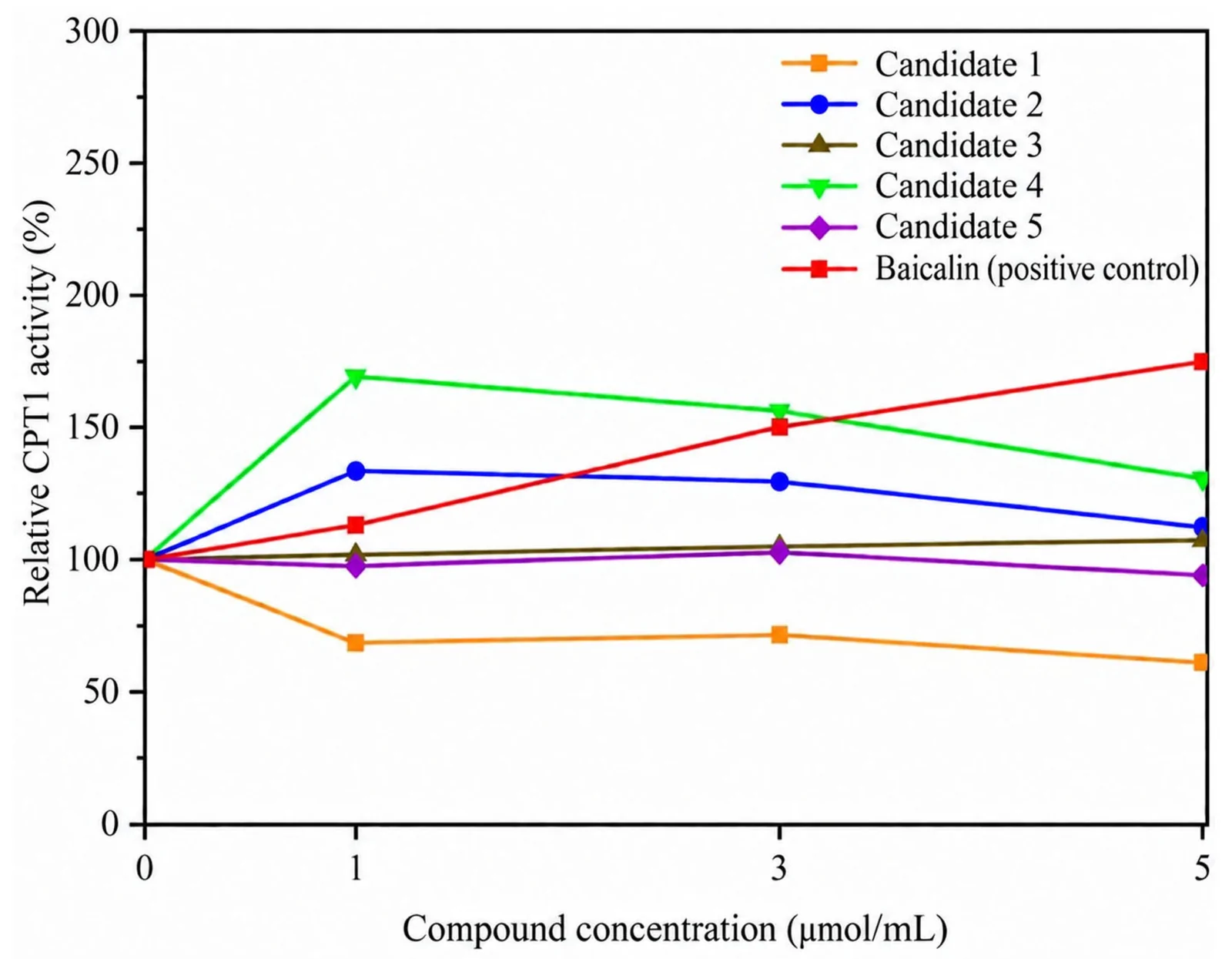
Preliminary in vitro CPT activity of candidates 1–5 and baicalin. Relative activity is expressed with the zero-concentration control set to 100%. Compound concentrations are given in μmol/mL.

To evaluate the preliminary in vitro activity of the screened compounds, CPT activity was assayed at 0, 1, 3, and 5 μmol/mL, using baicalin as a reference (Figure 7). Values were normalized to the zero-concentration control (set to 100%). Candidate 1 exhibited relative activities of approximately 69%, 72%, and 61% at 1, 3, and 5 μmol/mL, respectively. Candidate 2 remained above the control (~112–133%), whereas candidates 3 and 5 stayed close to the control (~102–107% and 94–103%, respectively). Candidate 4 reached approximately 169% at 1 μmol/mL and remained above the control at 3 and 5 μmol/mL (~156% and 131%). Baicalin reached approximately 175% at 5 μmol/mL.

## 3. Discussion

### 3.1. Local Conformational Analysis for CPT1A

Local conformational analysis characterized five flexible regions of membrane-embedded CPT1A. In residues 695–707, the 1 → 3 → 4 pathway accounted for 75.8% of the summed flux of the returned pathways between macrostates 1 and 4. This region includes Glu695 and Asp698, which contribute to the predicted baicalin binding pose. This link connects the local conformational analysis to a region examined during ligand screening.

Frame 467 connected the apo structural sampling to the subsequent docking analysis. Its occurrence in the late-stage RMSD plateau and the retained arrangement of most of the C-terminal catalytic domain supported its selection as the docking receptor. Hydrophobic packing, intramolecular contacts, and predicted cavities further characterized the structural environment used for screening. Together, these observations provided a structural rationale for selecting frame 467.

### 3.2. Catalytic and Regulatory Regions of CPT1A

The apo trajectory showed changes in the N-terminal and connector regions, as well as a relatively compact protein structure. This pattern can be considered in the context of the reported membrane-sensitive N-terminal regulatory switch [8] and previous membrane-curvature simulations of CPT1A [9]. Structural analysis of frame 467 identified hydrophobic packing, hydrogen-bond, and salt-bridge networks, and predicted cavities in the C-terminal region. These features motivated the use of this region for ligand screening.

Glu695 and Asp698 contribute to the predicted baicalin pose and lie within the selected C-terminal screening region. Candidate 2 also contacted Glu695 and exhibited the most favorable mean MM/PBSA estimate. Structural and simulation studies of carnitine acyltransferases describe catalytic channels and ligand-associated loop changes [22,23]. In the present models, the 695–707 region is therefore a specific site for examining how local conformations affect predicted ligand contacts.

### 3.3. Computational Prioritization of CPT1A Ligands

The docking and MM/PBSA analyses prioritized different candidates. Candidate 1 had the most favorable docking score, whereas candidate 2 had the most favorable mean MM/PBSA estimate. These differences reflect the distinct scoring procedures and structural inputs used in the two analyses. The candidate set provides defined chemical structures and predicted contacts for subsequent binding measurements. Previous CPT1A ligand-screening studies used mitochondrial membrane chromatography [17,19]. Chemoproteomic work identified baicalin as a CPT1A activator and linked its activity to improved lipid metabolism in diet-induced obese mice [24]. These findings motivate the search for additional CPT1A ligands. The present study contributes a receptor-selection and computational screening workflow. The reported roles of CPT1A in metabolic disease and cancer [2,3] provide broader biological context for the computational and preliminary activity findings. Earlier mitochondrial membrane chromatography studies identified baicalin, baicalein, wogonoside, wogonin, and resveratrol as CPT1A-associated modulators [17]; a later Twin Strep-tag CPT1A membrane chromatography study identified ononin, isoliquiritigenin, and aloe-emodin, which were reported to increase CPT1A activity in HepG2 cells [19]. In vitro, candidate 4 showed the highest relative activity at 1 μmol/mL, whereas candidate 1 remained below the zero-concentration control at all tested concentrations. These findings provide initial functional context for the computationally prioritized compounds.

The five local regions and the initial conformation for the docking were selected from MD trajectories. Additional apo trajectories and membrane compositions would test the sensitivity of the region selection and conformation choice [9,25,26]. Candidate 5 showed persistent fluctuations during the MD simulations, making it a priority for further sampling. The next experimental questions are whether the prioritized compounds bind to CPT1A and whether perturbing residues near 695–707 changes their binding or activity. These tests would connect the predicted contacts to ligand recognition and function.

### 3.4. Limitations

Despite the agreement between the computational predictions and the preliminary activity data, several caveats remain. Because no high-resolution experimental structure of full-length CPT1A is available, the starting model was AlphaFold-derived, and the conformational landscape sampled here may not capture other membrane compositions, longer timescales, or alternative apo states. Docking, SwissADME, and MM/PBSA analyses are approximate and do not explicitly account for all entropic, membrane, and kinetic contributions; the predicted binding modes and energies are therefore best regarded as a prioritization rather than proof of binding. The candidate-specific activity responses and their apparent agreement with the free-energy calculations will require confirmation through direct binding assays, independent replicates, dose–response measurements, and functional studies using purified or cellular CPT1A.

## 4. Materials and Methods

### 4.1. System Preparation

The initial structure of human CPT1A was downloaded from the AlphaFold Protein Structure Database v4 under accession AF-P50416-F1 (UniProt P50416) [27]. Predicted local confidence (pLDDT) is high across the C-terminal catalytic domain but low for the N-terminal regulatory region, consistent with the reported flexibility of that segment. The model was processed to remove hetero components and to add missing hydrogen atoms. The protein was oriented as an outer mitochondrial membrane (OMM) protein with its N-terminal regulatory region, two transmembrane helices, and cytosolic C-terminal catalytic domain arranged according to the reported CPT1A topology [6,7].

A membrane-embedded system was generated with CHARMM-GUI Membrane Builder [28]. The bilayer composition was chosen to approximate the OMM and was asymmetric between the outer and inner leaflets (Table 4) [26]. The protein–membrane complex was placed in a rectangular box with lateral dimensions of approximately 120 Å and a 22.5 Å water layer. K^+^ and Cl^−^ ions were added to neutralize the system and the salt concentration was adjusted to 0.15 mol/L. The final system contained approximately 150,000 atoms in total.

### 4.2. Molecular Dynamics (MD) Simulation

MD simulations were performed using GROMACS 2020.7 [29] with the CHARMM36m force field [30] under periodic boundary conditions. Energy minimization used steepest descent for up to 50,000 steps with a maximum-force tolerance of 1000 kJ mol^−1^ nm^−1^. The membrane-embedded apo system underwent two 125 ps NVT stages and four NPT stages of 125, 500, 500, and 500 ps, totaling 1.875 ns. The timestep was 1 fs for the first three stages and 2 fs thereafter. Equilibration used Berendsen temperature coupling [31] at 300 K and semi-isotropic Berendsen pressure coupling during NPT stages at 1 bar. The positional-restraint force constants were progressively reduced from 4000 to 50 for the protein backbone, 2000 to 0 for side chains, and 1000 to 0 kJ mol^−1^ nm^−2^ for lipids; dihedral restraints were also progressively reduced. Production MD lasted 1000 ns with a 2.0 fs timestep, Nosé-Hoover temperature coupling at 300 K, and semi-isotropic Parrinello-Rahman pressure coupling at 1 bar. No restraints were applied during the production simulations. Lennard-Jones forces were switched to zero between 1.0 and 1.2 nm, and PME electrostatics [32] used a 1.2 nm real-space cutoff. Bonds were constrained using LINCS [33]. Coordinates were saved every 100 ps.

Each candidate complex underwent 100 ps of NVT and 100 ps NPT equilibration, followed by 100 ns of production MD, using a 2.0 fs timestep. The ligand was free to move during the production MD simulations. V-rescale temperature coupling maintained 300 K, and isotropic Parrinello–Rahman pressure coupling maintained 1 bar during NPT equilibration and production. Coordinates were saved every 10 ps.

### 4.3. Markov State Modeling and Conformation Selection

Local Markov state models were constructed for five RMSF-enriched regions of CPT1A: residues 132–147, 235–247, 257–267, 420–440, and 695–707. Each region was analyzed separately using PyEMMA 2.5.12 [12]. Backbone φ and ψ dihedral angles were extracted using the add_backbone_torsions command, yielding 30, 24, 20, 40, and 24 input features for regions 1–5, respectively. We previously combined MD simulations, MSM analysis, and energetic calculations to investigate PPAR_γ_ conformational dynamics and guide ligand screening [15]. Related MD-MSM applications have characterized Src kinase activation intermediates and the coupling between protein conformational changes and ligand-binding kinetics [34,35].

The dihedral-angle features were discretized by k-means clustering with a maximum of 50 iterations and a clustering stride of 10. Bayesian MSMs were estimated from the resulting discrete trajectories using the region-specific microstate numbers and lag times listed in Table 5. Implied relaxation timescales were calculated for the four slowest processes with Bayesian uncertainty estimation. PCCA+ was then used to partition the microstates into four macrostates for transition-path analysis [13,14,15]. The selected lag times were 30, 20, 20, 45, and 30 frames for regions 1–5, respectively, corresponding to 60, 40, 40, 90, and 60 ns at the 2.0 ns sampling interval.

For structural clustering, an RMSD matrix was calculated for each region, and GROMOS clustering was performed using the region-specific cutoffs listed in Table 1 [16]. Frame 467 was used as the initial conformation for the docking. Its position along the backbone RMSD profile and its structural superposition with the initial CPT1A model were examined to characterize the selected conformation. Structural superimpositions of the four MSM macrostates and the GROMOS cluster representatives for each region are provided in Figures S1 and S2, respectively.

### 4.4. Docking and Drug-Likeness Prioritization

The selected frame was characterized with ProteinTools [36] to identify hydrophobic clusters, salt bridges, hydrogen-bond networks, and residue contacts. ProteinsPlus [37] and CavityPlus [38] were used independently to predict ligand-binding cavities. The C-terminal region identified by both tools and the predicted baicalin contacts guided the placement of the screening box. Baicalin was used as the reference ligand [18,19,24]. The final AutoDock box measured 38 × 42 × 34 Å and was centered at x = 16.667 Å, y = −0.444 Å, and z = 9.889 Å.

The ChemDiv (San Diego, CA, USA) collection dated 7 March 2024 contained 1,666,973 structures. Duplicate entries were removed, molecular formats standardized, missing hydrogens added, and protonation states optimized, yielding more than 1.23 million structures for docking. AutoDock Vina was used for batch screening [20]. Docking poses of the prioritized candidates were evaluated by jointly considering the Vina scores, the spatial fit within the selected pocket, and the compatibility of the ligand functional groups with the surrounding residues. The final poses selected on this basis were used for interaction analysis and complex preparation. Compounds were initially ranked by their best Vina scores, and the top 200 from the overall ranking were evaluated with SwissADME [21]. Five candidates were selected by jointly considering docking score, molecular weight, lipophilicity, hydrogen-bond capacity, predicted gastrointestinal absorption, blood–brain barrier permeation, and compliance with the Lipinski rule-of-five framework. The five CPT1A–candidate complexes were subjected to three independent 100 ns MD simulations.

### 4.5. Structural Analysis

The apo and complex trajectories were analyzed as separate datasets. Apo structural behavior was characterized using backbone root-mean-square deviation (RMSD), residue-wise root-mean-square fluctuation (RMSF), and radius of gyration (Rg). Protein structural behavior within each complex was characterized using RMSD, RMSF, Rg, and solvent-accessible surface area (SASA). PCA was performed independently for each complex using CPT1A C_α_ Cartesian coordinates. MDAnalysis [39] was used to read the trajectories and extract the coordinates. For each complex, the coordinates were arranged into a frame-by-coordinate matrix and mean-centered across frames. A separate PCA model was fitted using scikit-learn, and each trajectory was projected onto its own first two principal components. Secondary structure was assigned using the Dictionary of Secondary Structure of Proteins (DSSP) method [40].

### 4.6. Binding Free-Energy Analysis

MM/PBSA binding energy estimates (*ΔG_bind_*) were obtained using the molecular mechanics Poisson–Boltzmann surface area (MM/PBSA) method implemented in gmx_MMPBSA [41]. The energy terms are expressed as follows:

(1) $${\Delta G}_{bind}\;=\;{\Delta G}_{Complex}\;-\;{\Delta G}_{Receptor}\;-\;{\Delta G}_{ligand}$$

(2) $${\Delta G}_{bind}={\Delta E}_{MM}+{\Delta G}_{PB}+{\Delta G}_{Surf}-T\Delta S$$

where *ΔE_MM_* is the gas-phase molecular mechanical energy, comprising electrostatic (*ΔE_ele_*) and van der Waals (*ΔE_vdW_*) contributions. The solvation free energy includes polar (*ΔG_PB_*) and nonpolar (*ΔG_Surf_*) components. The *–TΔS* represents the entropic contribution. Frames from 80–100 ns were used for the five candidate complexes and the baicalin reference simulation. More negative total values were used to rank the calculated energetics of the candidate complexes.

### 4.7. In Vitro CPT Activity Assay

Enzyme-containing samples were prepared as mitochondrial membrane suspensions from HepG2 cells using a procedure similar to the previously described CPT1A activity workflow [17,19]. Cells were harvested by centrifugation at 1000× *g*, washed once with phosphate-buffered saline (PBS; Sigma-Aldrich, St. Louis, MO, USA), and mitochondria were isolated by gradient centrifugation under ice-cold conditions. The mitochondrial fraction was sonicated to obtain the membrane suspension. CPT activity was measured with a commercial microplate assay kit (catalog no. ml301015; Shanghai Enzyme-linked Biotechnology Co., Ltd., Shanghai, China). Each well contained 10 μL of sample, 220 μL of Reagent 5, and 10 μL of Reagent 6. Absorbance at 412 nm was recorded at 20 and 140 s, and the change over 2 min was used to calculate activity. Candidates 1–5 and baicalin were assessed at 0, 1, 3, and 5 μmol/mL. Relative activity was normalized to the zero-concentration control (100%).

## 5. Conclusions

Carnitine palmitoyltransferase 1A (CPT1A), the rate-limiting enzyme of mitochondrial fatty acid β-oxidation, has emerged as a target for metabolic disease and cancer, yet its membrane-bound dynamics remain difficult to capture from static models alone. We therefore embedded an AlphaFold-derived human CPT1A model in the membrane environment and performed the all-atom MD simulations, followed by local MSM/transition-path analysis, GROMOS clustering, docking, and MM/PBSA calculations.

The apo trajectories retained a compact C-terminal catalytic domain, whereas the N-terminal and connector regions underwent larger rearrangements. Frame 467, taken from the late-stage RMSD plateau, captured this mixed stability. Screening more than 1.23 million ChemDiv compounds against frame 467 yielded five candidates. The MM/PBSA values of candidate 2 are the most negative (−25.99 ± 1.85 kcal/mol), followed by candidates 4, 1, 3, and 5 (−18.99, −18.36, −15.47, and −11.01 kcal/mol), compared with baicalin (−13.28 ± 0.42 kcal/mol). Preliminary activity measurements were partially consistent with the computational trends, but remain exploratory. Inspired by these findings, future work can focus on: (1) experimentally testing candidate 2 and candidate 1 for CPT1A binding and activity; (2) optimizing the candidates to strengthen contacts with Glu695/Asp698 and the C-terminal pocket. These results provide a dynamic structural basis for CPT1A ligand discovery and a practical route for receptor selection in membrane proteins.

## Figures and Tables

**Table 1:** Dominant local transition pathways and GROMOS clustering results.

Region	Dominant Pathway	Relative Pathway Flux (%)	GROMOS Cutoff (Å)	Clusters	Representative Frame
1	1 → 3 → 4	56.8%	1.50	4	467
2	1 → 4	68.1%	1.50	3	367
3	1 → 2 → 3 → 4	68.9%	0.80	4	444
4	1 → 4	65.2%	1.50	3	496
5	1 → 3 → 4	75.8%	2.00	3	467

Residues 132–147 (region 1), 235–247 (region 2), 257–267 (region 3), 420–440 (region 4), and 695–707 (region 5).

**Table 2:** Docking and predicted drug-likeness properties of the five candidates.

Candidate	ChemDiv ID	Formula	Docking Score (kcal/mol)	MW (Da)	LogP	HBD	HBA	GI/BBB
1	8011-0661	C31H26N2O	−11.20	442.55	5.31	2	2	High/Yes
2	3257-4808	C34H22N4	−11.00	486.57	6.87	0	4	Low/No
3	6703-2477	C25H16N2O2	−10.50	376.41	4.39	1	4	High/Yes
4	7610-1541	C28H22N4O	−10.40	430.50	4.77	0	4	High/Yes
5	4826-0414	C24H16N6O3	−10.40	436.42	3.15	1	9	High/No

MW, molecular weight; LogP, logarithm of the octanol/water partition coefficient; HBD, hydrogen-bond donor count; HBA, hydrogen-bond acceptor count; GI, predicted gastrointestinal absorption (High/Low); BBB, predicted blood–brain barrier permeation (Yes/No). In the GI/BBB column, absorption is listed first and barrier permeation second.

**Table 3:** MM/PBSA energy components for baicalin and the five candidates.

Ligand	*ΔE_vdW_*	*ΔE_ele_*	*ΔG_PB_*	*ΔG_Surf_*	*ΔG_bind_*
Baicalin	−24.78 ± 0.67	−3.53 ± 0.28	17.41 ± 0.50	−2.38 ± 0.03	−13.28 ± 0.42
Candidate 1	−37.63 ± 0.99	−4.64 ± 0.86	27.86 ± 0.49	−3.95 ± 0.01	−18.36 ± 1.40
Candidate 2	−35.81 ± 1.20	−1.33 ± 1.18	14.92 ± 0.77	−3.78 ± 0.02	−25.99 ± 1.85
Candidate 3	−18.52 ± 0.79	−28.02 ± 0.45	33.26 ± 0.53	−2.20 ± 0.01	−15.47 ± 1.05
Candidate 4	−25.04 ± 1.48	−2.24 ± 0.72	11.23 ± 0.19	−2.95 ± 0.03	−18.99 ± 1.66
Candidate 5	−15.71 ± 2.95	−6.76 ± 0.14	13.00 ± 1.49	−1.53 ± 0.85	−11.01 ± 3.42

All values are given in kcal/mol, and behind “±” are their standard deviations (S.D.).

**Table 4:** Leaflet-specific lipid composition of the asymmetric mitochondrial outer membrane-mimetic bilayer.

Lipid Species	Outer Leaflet (%)	Inner Leaflet (%)
POPC	6	6
PLPC	18	18
SAPC	30	30
POPE	4	2
PLPE	9	5
SAPE	25	13
SAPI	4	22
SAPS	1	3
POPA	1	1
TLCL1	2	0
Total	100	100

**Table 5:** Region-specific parameters used for local Markov state modeling.

Region	Residues	Atoms	Dihedral Features	Microstates (k)	Lag (Frames)
1	132–147	257	30	25	30
2	235–247	234	24	15	20
3	257–267	190	20	15	20
4	420–440	302	40	30	45
5	695–707	205	24	15	30
‘Atoms’ denotes the total number of atoms in each regional input structure. ‘Dihedral Features’ denotes the number of backbone torsion angles used for clustering. One lag frame corresponds to 2.0 ns. Transition path theory was applied between source macrostate 1 and target macrostate 4. The reactive flux network was coarse-grained over the four macrostates and decomposed into pathways using a cumulative flux target of 0.99. The relative flux of pathway (*r*) was calculated as:					
$p_{r}=\frac{f_{r}}{\sum_{s\in P}f_{s}}$					
where *f_r_* is the flux assigned to pathway *r*, and 𝒫 is the set of pathways returned by the decomposition. The relative pathway fluxes are expressed as percentages.					

## Data Availability

The simulation parameters, analysis procedures, and summarized results are reported in the main text and source thesis. Additional trajectory and screening data may be requested from the corresponding author.

## Supplementary Materials

The following supporting information can be downloaded at: https://www.mdpi.com/article/10.3390/ijms27198840/s1.
