# Supplementary material for: Conformational Sampling of CPT1A Enables Dynamic Structure-Based Ligand Screening
Source: Int J Mol Sci. 2026 Oct 3;27(19):8840. doi: 10.3390/ijms27198840 (PMC13664960; doi:10.3390/ijms27198840)
Supplement: Supplementary file 1 [file ijms-27-08840-s001.zip › ijms-4564992-supplementary.pdf]

Supplementary Materials

Conformational Sampling of CPT1A Enables Dynamic Structure-Based Ligand Screening

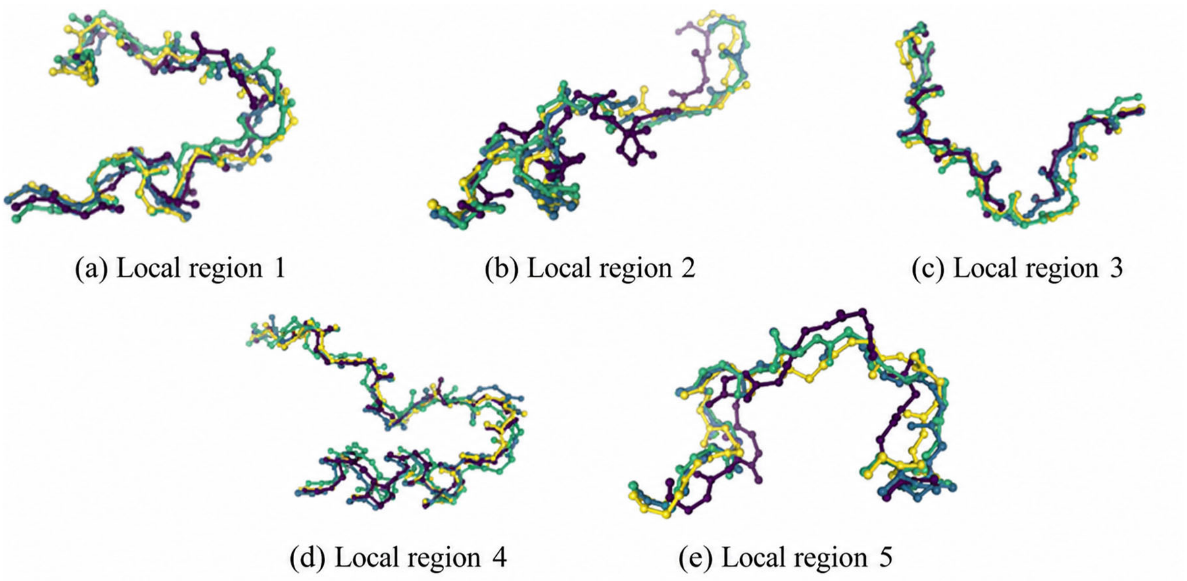

Figure S1. Superposition of the four MSM macrostate conformations for the five local regions of CPT1A. Panels (a–e) correspond to regions 1–5, respectively; colors distinguish the four macrostates.

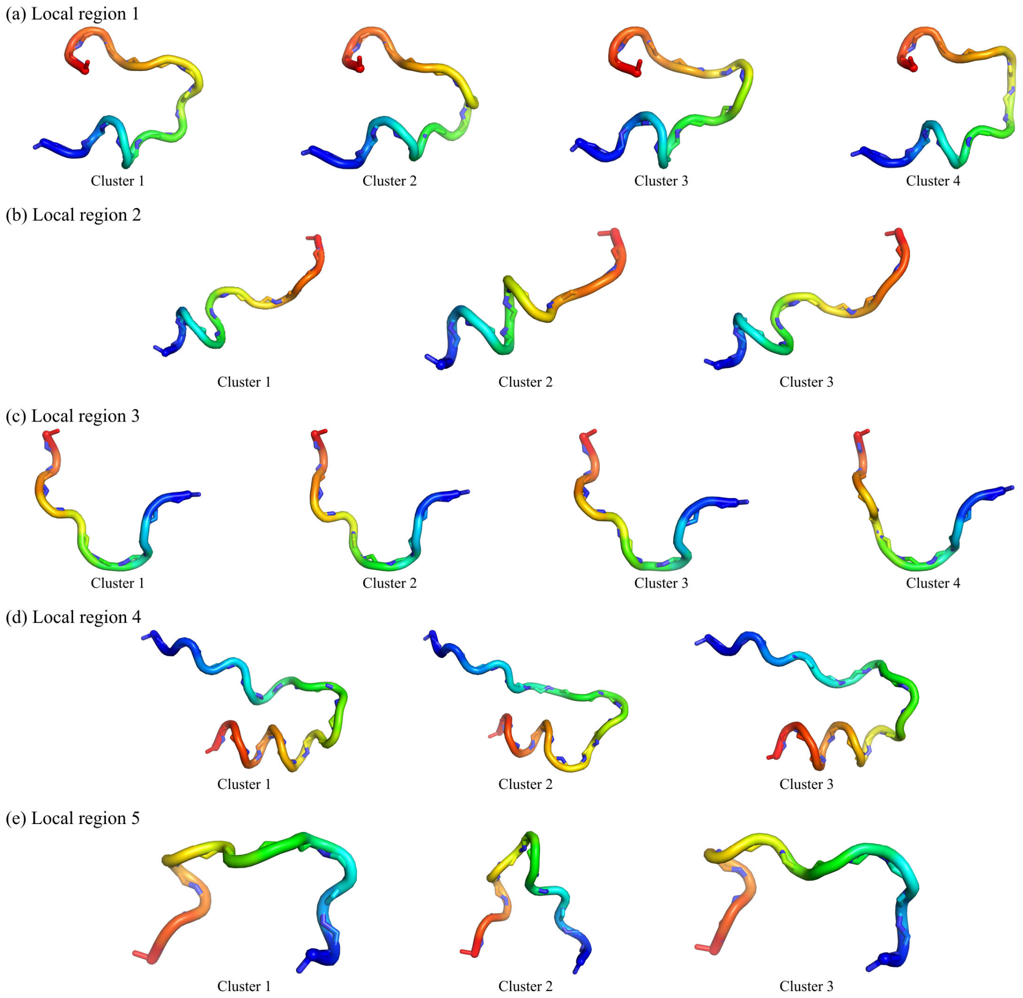

Figure S2. GROMOS cluster conformations for the five local regions of CPT1A. Regions 1–5 contained 4, 3, 4, 3, and 3 clusters, respectively.

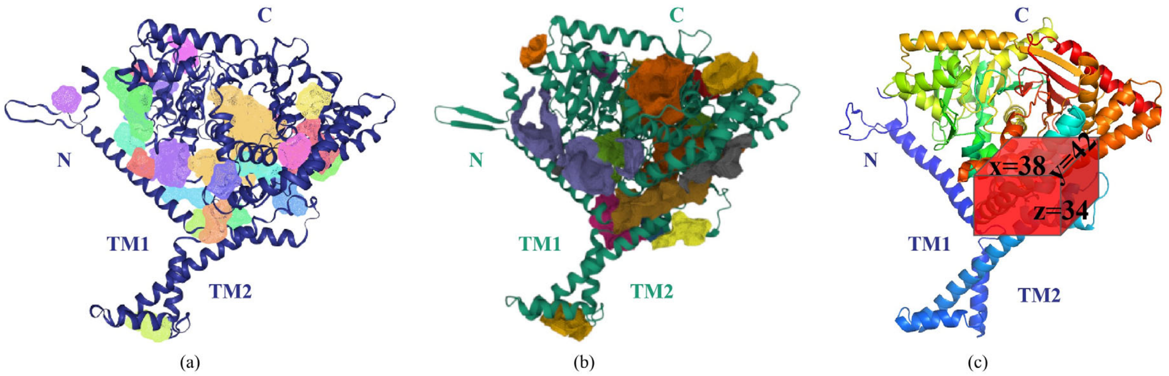

Figure S3. Binding-pocket prediction and docking-box definition for the representative CPT1A conformation (frame 467). (a) The 25 cavities predicted by ProteinsPlus[1]. (b) The 21 cavities predicted by CavityPlus[2]. (c) The docking box used for virtual screening (x = 38 Å, y = 42 Å, and z = 34 Å).

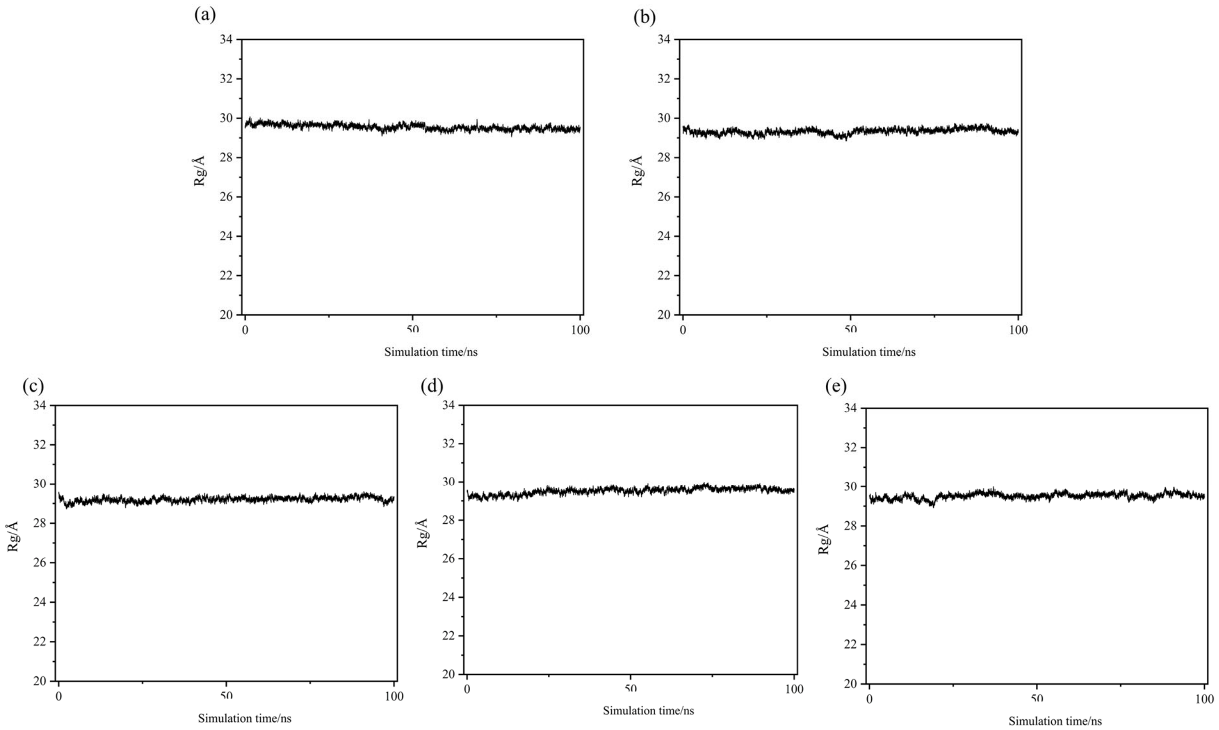

Figure S4. Total radius-of-gyration (Rg) profiles of CPT1A complexes with the five candidate compounds during 100 ns MD simulations. Panels (a–e) correspond to candidates 1–5, respectively.

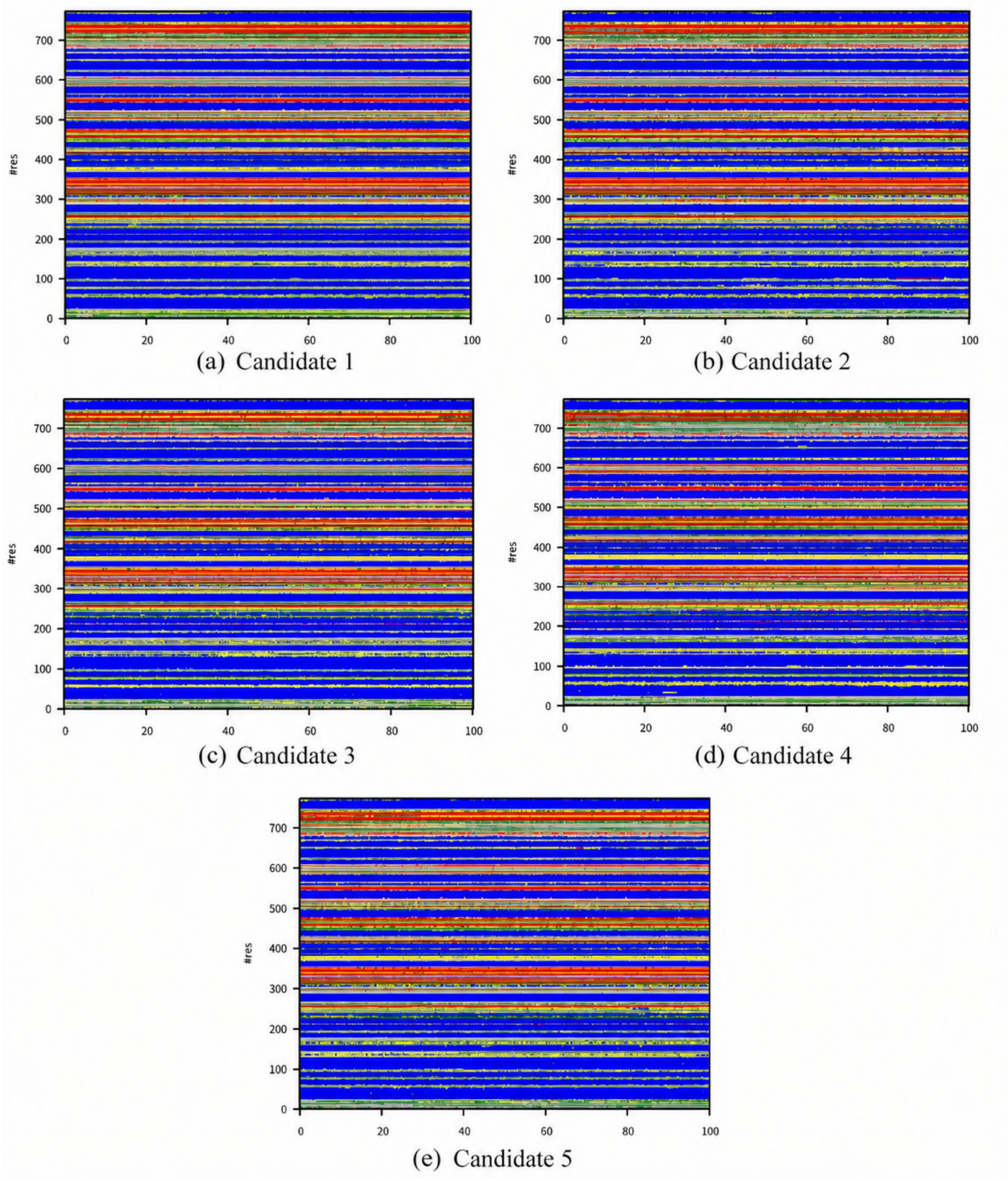

Figure S5. Time evolution of the secondary structures of CPT1A complexes with candidates 1–5 during 100 ns MD simulations. Panels (a–e) correspond to candidates 1–5, respectively. Blue, α-helix; red, β-sheet; yellow, turn; light gray, coil.

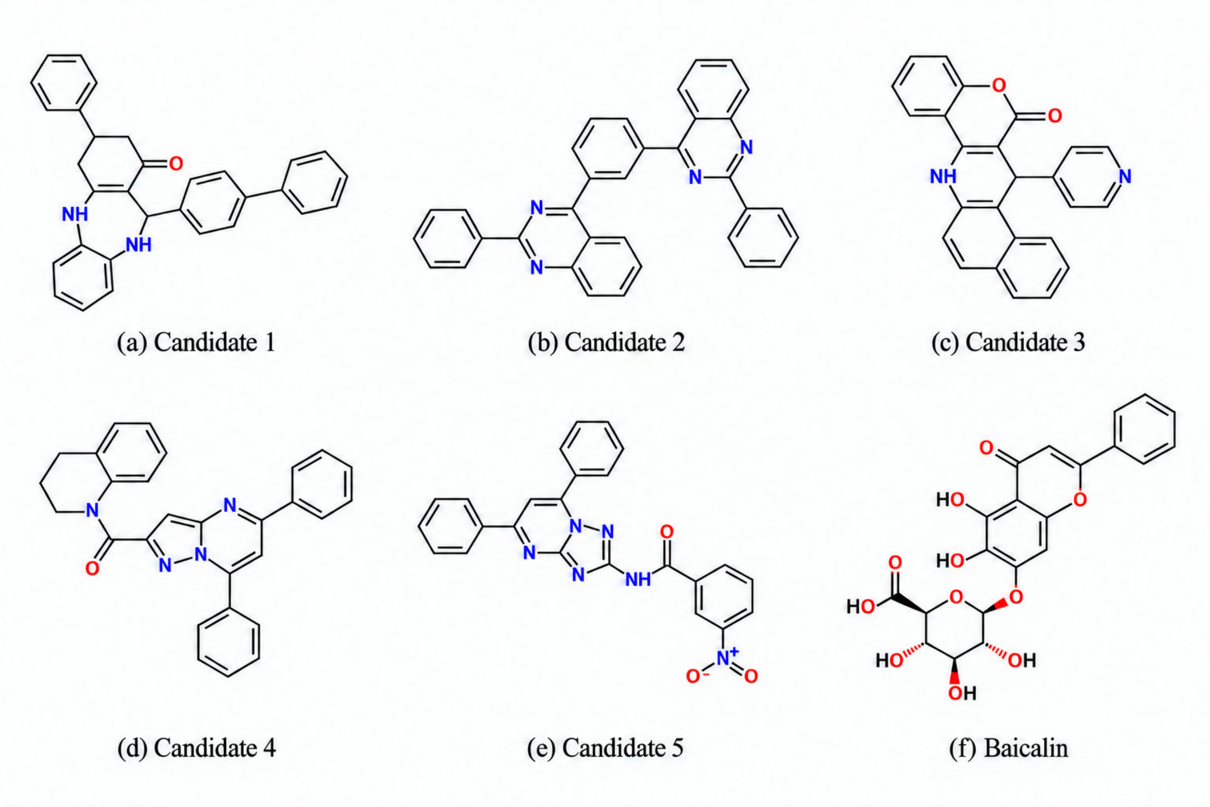

Figure S6. Two-dimensional chemical structures of candidates 1–5 (a–e) and baicalin (f). Nitrogen and oxygen atoms are shown in blue and red, respectively.

References

1. Schöning-Stierand, K.; Diedrich, K.; Fährrolfes, R.; Flachsenberg, F.; Meyder, A.; Nittinger, E.; Steinegger, R.; Rarey, M., ProteinsPlus: Interactive Analysis of Protein–Ligand Binding Interfaces. *Nucleic Acids Res.* **2020,** 48, (W1), W48-W53.
2. Xu, Y.; Wang, S.; Hu, Q.; Gao, S.; Ma, X.; Zhang, W.; Shen, Y.; Chen, F.; Lai, L.; Pei, J., CavityPlus: A Web Server for Protein Cavity Detection with Pharmacophore Modelling, Allosteric Site Identification and Covalent Ligand Binding Ability Prediction. *Nucleic Acids Res.* **2018,** 46, (W1), W374-W379.
